# Pulmonary Veno-Occlusive Disease Presenting as Congenital Heart Disease–Associated Pulmonary Hypertension

**DOI:** 10.1016/j.jaccas.2026.109910

**Published:** 2026-08-20

**Authors:** Takanori Suzuki, Satoru Kawai, Satona Tanaka, Hidetoshi Uchida, Hiromu Ukai, Yoji Nomura, Hiroyuki Katsuragawa, Jun-ichi Kawada, Shuichiro Yoshida

**Affiliations:** a Department of Pediatrics, Kariya Toyota General Hospital, Aichi, Japan; b Department of Pediatric Cardiology, Aichi Children's Health and Medical Center, Aichi, Japan; c Department of Pediatrics, School of Medicine, Fujita Health University, Aichi, Japan; d Department of Thoracic Surgery, Kyoto University Hospital, Kyoto, Japan; e Department of Diagnostic Pathology, Kyoto University Hospital, Kyoto, Japan

**Keywords:** lung transplantation, pulmonary hypertension, pulmonary veno-occlusive disease, ventricular septal defect

## Abstract

**Background:**

Pulmonary veno-occlusive disease (PVOD) is a rare cause of pulmonary hypertension rarely linked to congenital heart disease (CHD).

**Case Summary:**

An infant with a ventricular septal defect developed pulmonary hypertension. At 3 months, cardiac catheterization showed operable hemodynamics with preserved pulmonary vasoreactivity. Pulmonary artery banding was performed. Despite tadalafil, macitentan, and prostacyclin, pulmonary vascular resistance increased without pulmonary edema. Lung perfusion scintigraphy demonstrated perfusion defects, prompting transplantation. At 2 years and 10 months, he underwent bilateral lung transplantation with intracardiac repair. The explanted lungs demonstrated fibrous intimal thickening of pulmonary venules and hemosiderin deposition, indicating PVOD.

**Discussion:**

PVOD may coexist with CHD and mimic CHD-associated pulmonary arterial hypertension, without pulmonary edema during vasodilator therapy.

**Take-Home Messages:**

PVOD should be considered in children with CHD developing progressive pulmonary hypertension despite favorable hemodynamics and preserved vasoreactivity. The absence of pulmonary edema during vasodilator therapy does not exclude PVOD.

## Visual Summary

**Figure:**
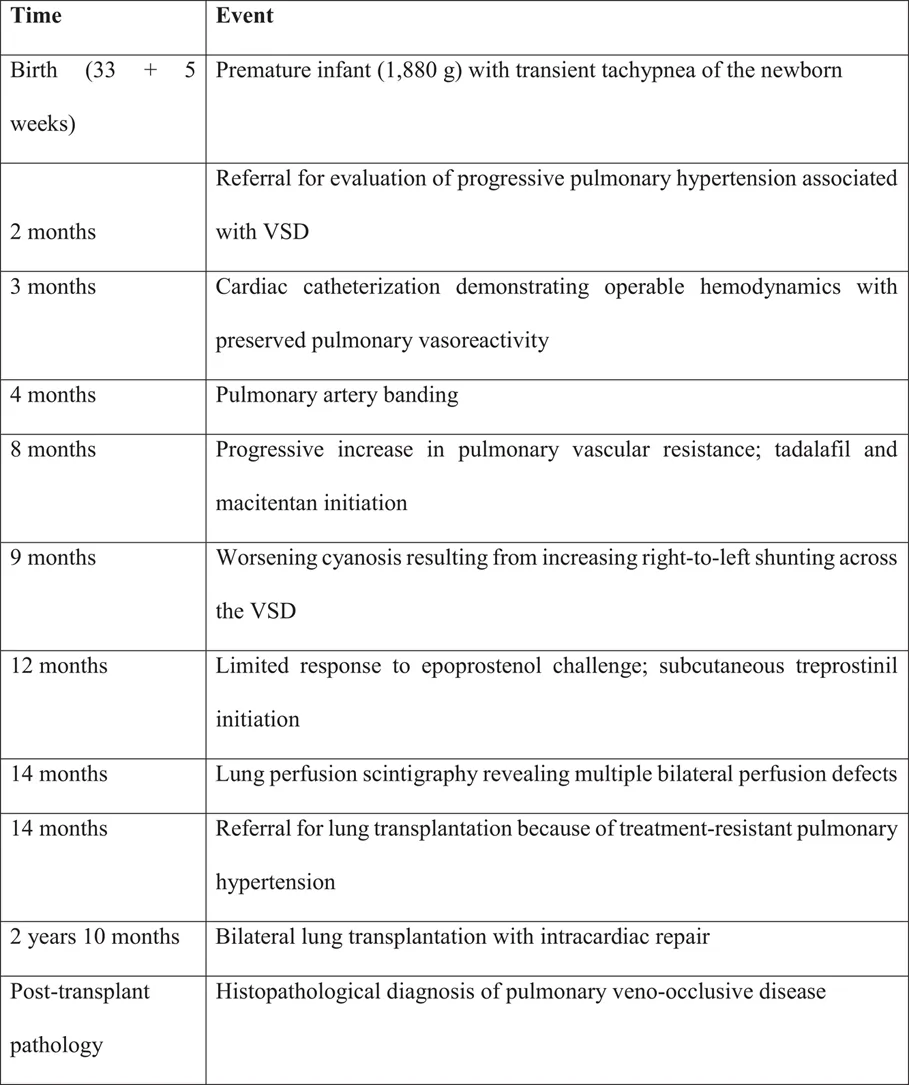

## History of Presentation

A 2-month-old male infant was referred for evaluation of progressive pulmonary hypertension associated with a ventricular septal defect (VSD). During follow-up, echocardiography demonstrated that left-to-right shunt velocity progressively decreased across the VSD from 2.8 to 1.5 m/s, raising concern for increasing pulmonary arterial pressure. Thus, he was referred to our institution for further evaluation (Figures 1A and 1B).

**Figure 1:**
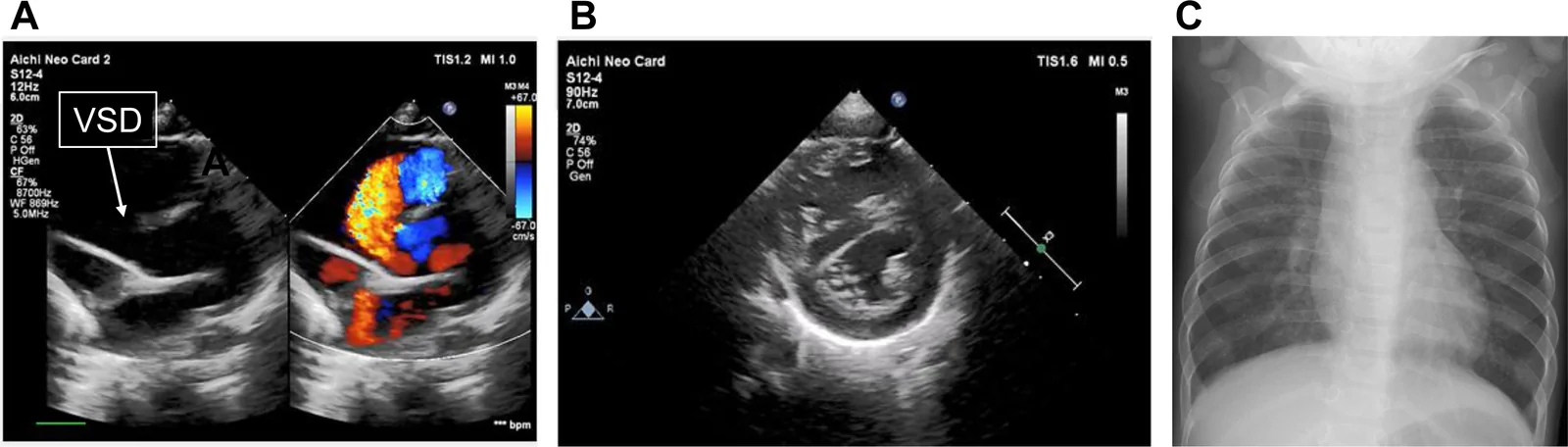
Echocardiographic and Radiographic Findings (A) Parasternal short-axis view demonstrates a 6.5-mm perimembranous ventricular septal defect with left-to-right shunting. (B) Parasternal short-axis view shows interventricular septal flattening with left ventricular compression caused by right ventricular pressure overload, indicating pulmonary hypertension. (C) Chest radiograph obtained during combination pulmonary vasodilator therapy, including subcutaneous treprostinil (24 ng/kg/min), revealed no pulmonary edema despite progressive pulmonary vascular disease. VSD = ventricular septal defect.

## Past Medical History

The patient was born at 33 weeks and 5 days of gestation via cesarean delivery secondary to hypertensive disorders of pregnancy, with a weight of 1,880 g. He was supplemented with oxygen for 3 days to manage transient tachypnea of the newborn; he did not require endotracheal intubation or mechanical ventilation. Shortly after birth, a perimembranous VSD was detected and managed with diuretics and digoxin. There was no family history of pulmonary hypertension, pulmonary veno-occlusive disease (PVOD)/pulmonary capillary hemangiomatosis (PCH), unexplained respiratory disease, or premature cardiopulmonary death.

## Differential Diagnosis

The differential diagnosis included congenital heart disease (CHD)–associated pulmonary arterial hypertension (PAH) secondary to VSD—the most likely diagnosis at presentation. Other potential causes of pulmonary hypertension (pulmonary vein stenosis, cor triatriatum, scimitar syndrome, chronic lung disease of prematurity, connective tissue disease–associated PAH) were also considered. Given that pulmonary vascular resistance progressively increased despite appropriate management, PVOD was considered as a less common but important differential diagnosis.

## Investigations

Computed tomography ruled out pulmonary vein stenosis, cor triatriatum, scimitar syndrome, significant lung hypoplasia, and chronic lung disease as causes of pulmonary hypertension in this case. Serologic evaluation for connective tissue disease, including antinuclear antibodies and other autoimmune markers, was unremarkable. Cardiac catheterization at 3 months of age demonstrated a mean pulmonary arterial pressure of 36 mm Hg, pulmonary arterial wedge pressure of 8 mm Hg, pulmonary vascular resistance index (PVRi) of 3.14 WU·m^2^; and pulmonary-to-systemic blood flow ratio of 1.6. Pulmonary vasoreactivity was preserved, with no pulmonary venous obstruction detected. Despite intervention, pulmonary vascular disease progressed. At 8 months of age, PVRi increased (9.9 WU·m^2^) but then improved with oxygen and inhaled nitric oxide administration (4.0 WU·m^2^). At 9 months of age, the patient exhibited decreased pulmonary-to-systemic blood flow ratio (0.68), with worsening cyanosis. During epoprostenol challenge at 12 months of age, pulmonary hemodynamics modestly improved. At 14 months of age, PVRi remained elevated (7.0 WU·m^2^) despite treprostinil therapy (Figure 2). On lung perfusion scintigraphy, multiple bilateral subsegmental perfusion defects were detected (Figure 3A). Genetic testing was performed at the Kazusa DNA Research Institute using a targeted next-generation sequencing panel including *BMPR2*, *ACVRL1*, *ENG*, *SMAD9*, *CAV1*, *KCNK3*, and *EIF2AK4.* The analysis assessed single-nucleotide variants and small insertions/deletions but did not evaluate copy-number variants. No pathogenic or likely pathogenic variants were identified.

**Figure 2:**
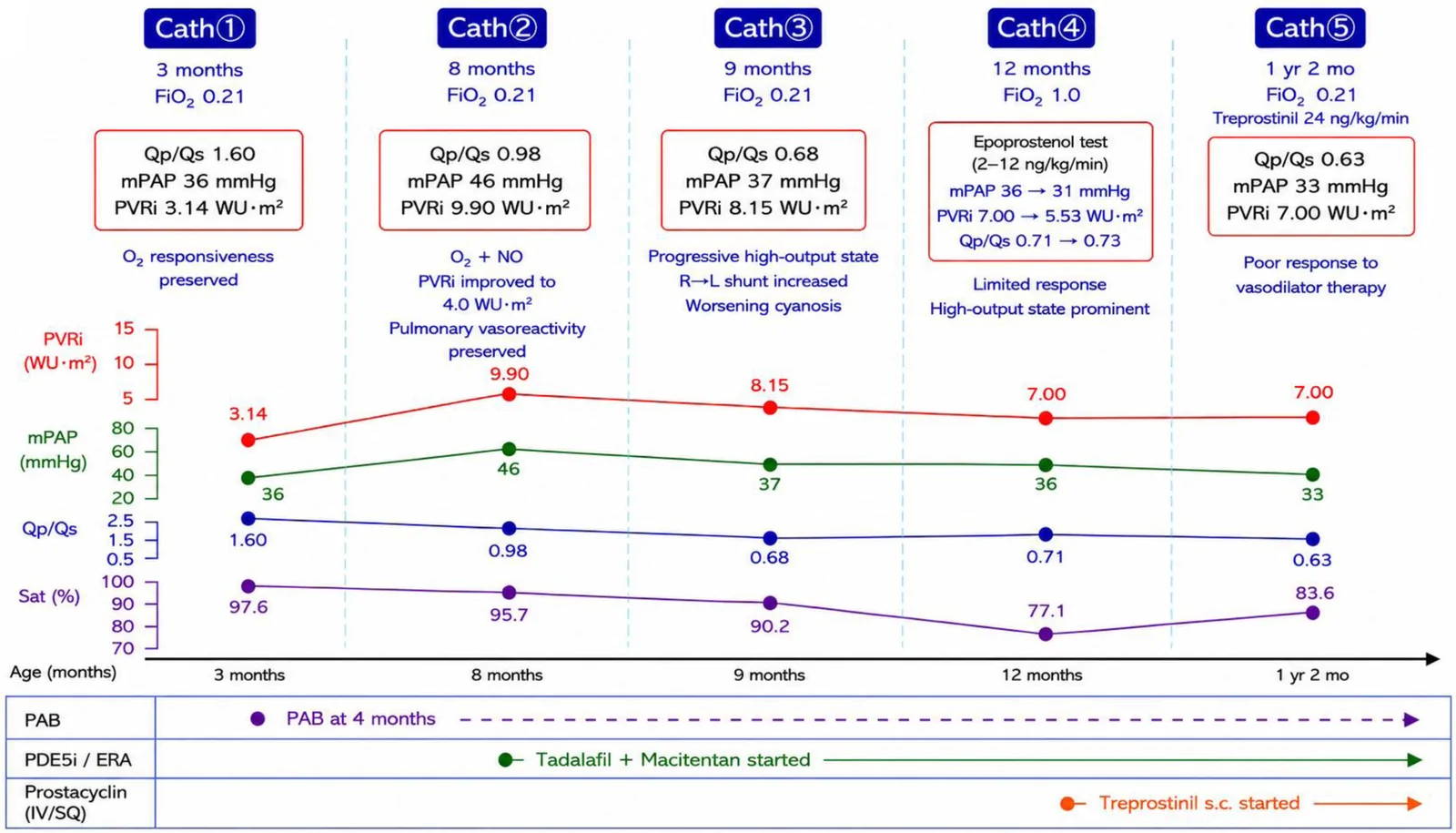
Serial Hemodynamic Changes and Therapeutic Interventions Serial cardiac catheterization findings suggest pulmonary vascular disease progression despite escalation of the pulmonary vasodilator therapy. At 3 months of age, the hemodynamics were considered operable, with preserved pulmonary vasoreactivity (PVRi: 3.14 WU·m^2^, Qp/Qs: 1.60). Following pulmonary artery banding at 4 months of age, pulmonary vascular resistance progressively increased. Although pulmonary vasoreactivity remained intact at 8 months of age, subsequent catheterizations demonstrated persistent PVRi elevation, worsening right-to-left shunting across the ventricular septal defect, and limited response to prostacyclin therapy. Despite combination treatment with tadalafil, macitentan, and treprostinil, pulmonary vascular disease progression persisted. ERA = endothelin receptor antagonist; Fio_2_ = fraction of inspired oxygen; IV = intravenous; L = left; mPAP = mean pulmonary arterial pressure; NO = nitric oxide; PAB = pulmonary artery banding; PDE5i = phosphodiesterase type 5 inhibitor; PVRi = pulmonary vascular resistance index; Qp/Qs = pulmonary-to-systemic blood flow ratio; R = right; Sat = saturation; SQ = subcutaneous.

**Figure 3:**
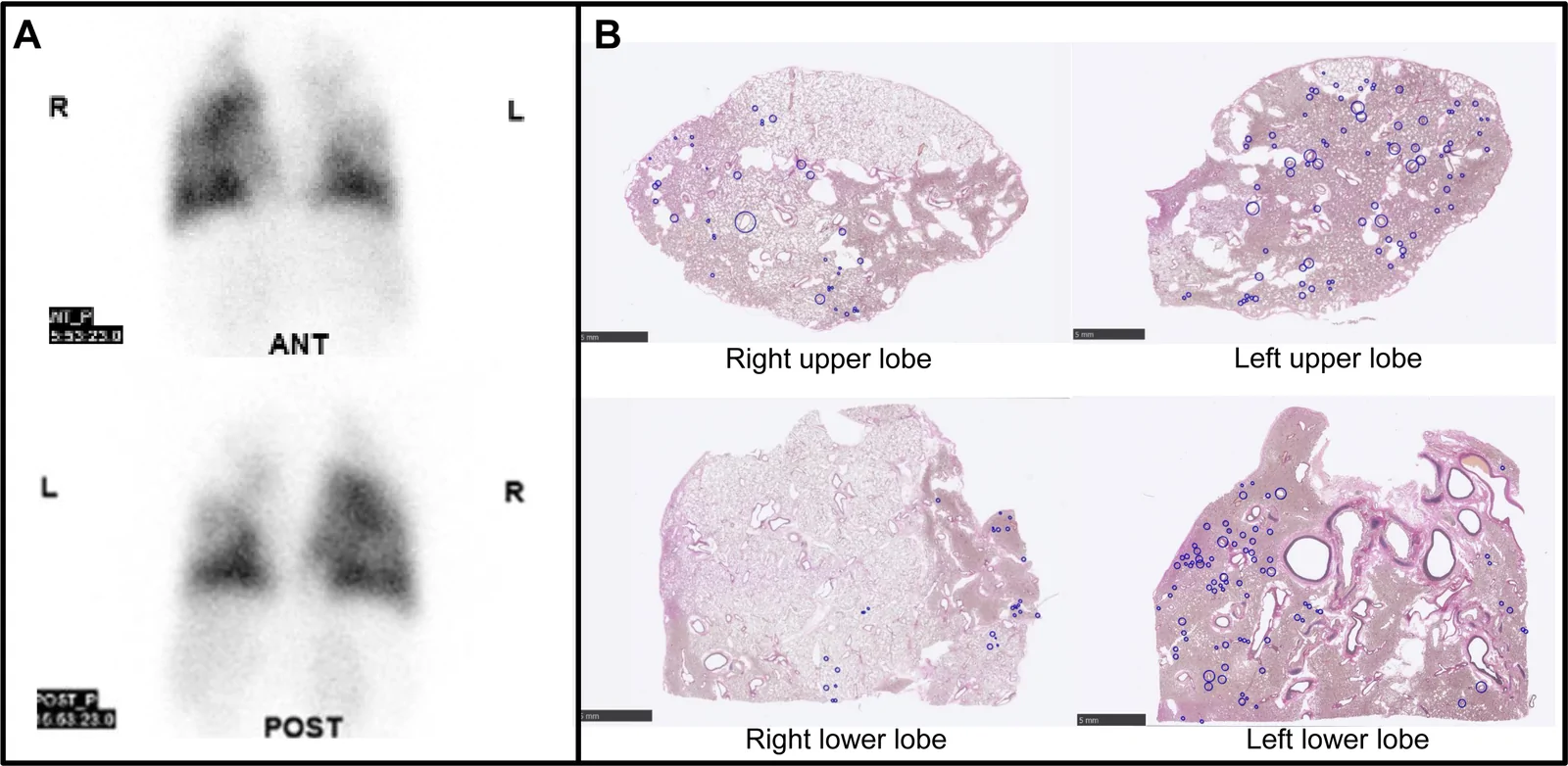
Correlation Between Lung Perfusion Scintigraphy and Distribution of PVOD Lesions (A) Lung perfusion scintigraphy demonstrated multiple bilateral subsegmental perfusion defects, suggesting heterogeneous and diffuse pulmonary vascular involvement. (B) Gross examination of the explanted lungs revealed areas with prominent pulmonary venous occlusive lesions (blue circles). The distribution of these lesions corresponded to the perfusion abnormalities identified on pretransplant lung perfusion scintigraphy, supporting the presence of regionally heterogeneous PVOD. ANT = anterior; L = left; POST = posterior; PVOD = pulmonary veno-occlusive disease; R = right.

## Management

Considering the hemodynamic findings, the patient underwent pulmonary artery banding at 4 months of age. Given preserved pulmonary vasoreactivity, tadalafil and macitentan were initiated at 8 months of age. Pulmonary hypertension progressed; thus, prostacyclin therapy was added and was escalated to subcutaneous treprostinil. Despite this combination pulmonary vasodilator therapy, pulmonary vascular disease continued to worsen. Notably, pulmonary edema did not occur, as there was no new or worsening respiratory distress, increased oxygen requirement, or radiographic evidence of pulmonary congestion on serial chest radiographs (Figure 1C). Given the patient's progressive and treatment-resistant pulmonary hypertension, he was referred for lung transplantation.

## Outcome and Follow-Up

At 2 years and 10 months of age, the patient underwent bilateral lung transplantation with intracardiac repair. Subsequently, the explanted lungs were histopathologically examined, revealing marked luminal narrowing resulting from diffuse fibrous intimal thickening with occlusion of small pulmonary veins and venules (Figures 4A and 4B). Conversely, advanced pulmonary arterial remodeling was limited, with only mild arterial changes identified despite severe pulmonary hypertension (Figure 4C). In addition, hemosiderin-laden macrophages accumulated within the alveolar spaces, indicating chronic pulmonary venous congestion (Figure 4D). Meanwhile, the pulmonary venous lesions were heterogeneously distributed throughout the lungs, corresponding to the regional perfusion abnormalities observed on lung perfusion scintigraphy. For quantitative pathological assessment, 4 tissue samples were obtained from each of the right upper, right lower, left upper, and left lower lobes. The glass slides were digitized using a NanoZoomer system (Hamamatsu Photonics). The evaluated tissue area and number of pulmonary venous occlusive lesions were measured, and lesion density was calculated as the number of lesions per square centimeter of evaluated tissue. Lesion density was highest in the left upper lobe (19.73 lesions/cm^2^), followed by the right upper lobe (12.04 lesions/cm^2^), left lower lobe (8.71 lesions/cm^2^), and right lower lobe (4.55 lesions/cm^2^), demonstrating a heterogeneous lobar distribution (Figure 3B). The most prominent perfusion abnormality on lung perfusion scintigraphy was also observed in the left upper lobe (Figure 3A). Detailed measurements are provided in Supplemental Table 1. Collectively, these findings were diagnostic of PVOD and indicated that pulmonary venous pathology, rather than pulmonary arterial disease, was the main mechanism underlying pulmonary hypertension in this case. Postoperatively, no untoward events occurred, with no reintubation or prolonged mechanical ventilatory support required. Subsequently, the patient was discharged home. At the most recent follow-up, he remained stable with satisfactory post-transplant functional status.

**Figure 4:**
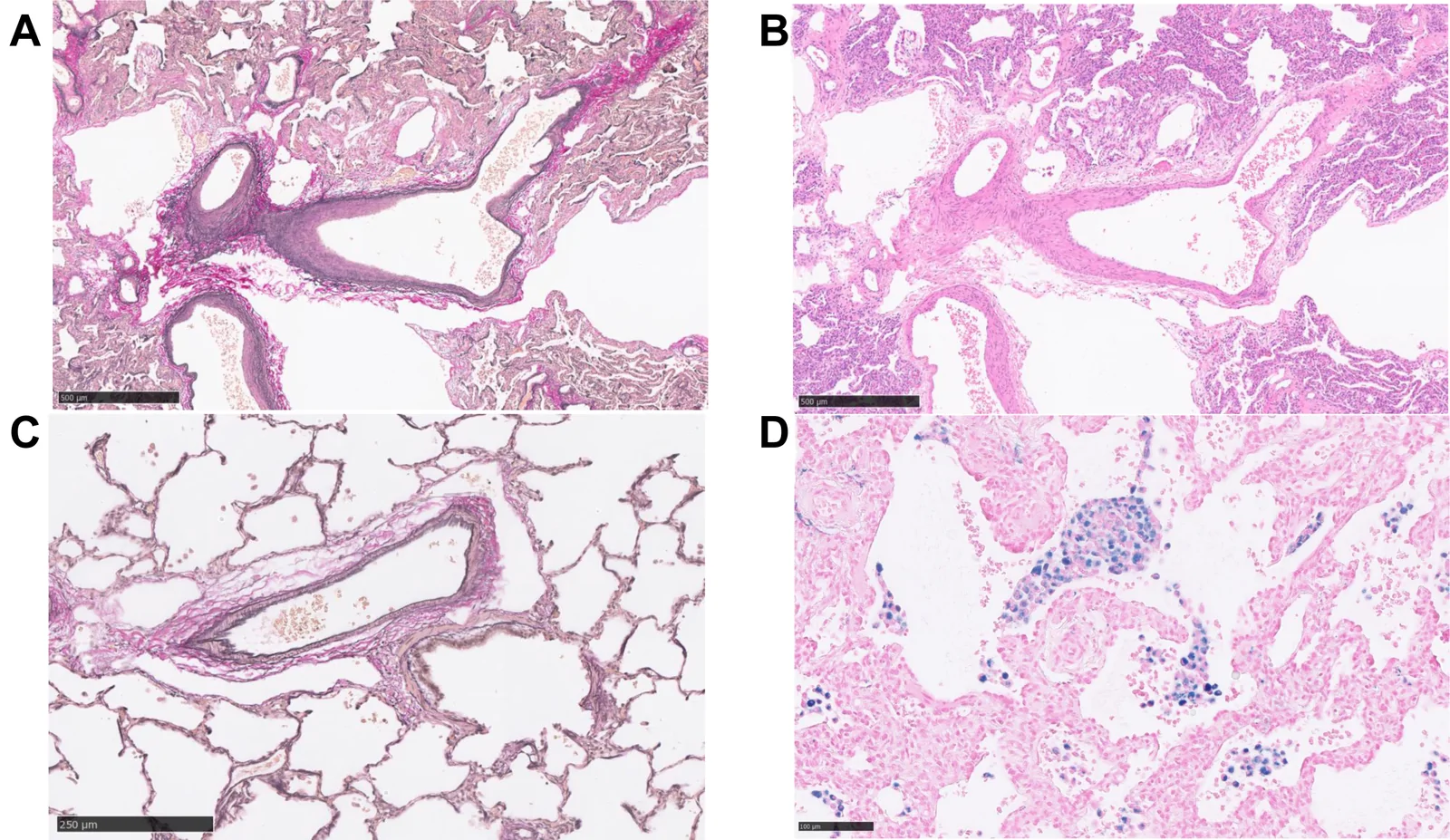
Histopathological Findings of the Explanted Lungs (A) Elastica van Gieson staining demonstrated marked fibrous intimal thickening of a pulmonary vein, with luminal narrowing of >30%. (B) Hematoxylin and eosin staining confirms prominent fibrous intimal thickening of the pulmonary venous wall. (C) Elastica van Gieson staining of a pulmonary artery revealed no significant arterial wall fibrosis or medial hypertrophy. (D) Berlin blue staining showed hemosiderin-laden macrophages, suggesting chronic pulmonary venous congestion. The predominance of pulmonary venous remodeling without advanced pulmonary arterial remodeling supported the diagnosis of pulmonary veno-occlusive disease.

## Discussion

PVOD is a rare cause of pulmonary hypertension characterized by progressive obstruction of the pulmonary veins and venules.^1^ The seventh World Symposium on Pulmonary Hypertension reclassified PVOD within group 1 PAH (group 1.5) to emphasize the shared clinical and hemodynamic characteristics between PVOD and PAH despite their distinct pathological substrates.^2^ Given that definitive diagnosis generally requires histopathological confirmation, many patients are diagnosed only after lung transplantation or autopsy.^3^^,^^4^

PVOD coexisting with CHD is exceptionally rare. Chida-Nagai et al^5^ reported a series of patients with CHD-associated PVOD/PCH, many of whom had chromosomal abnormalities or syndromic conditions. In the present case, no syndromic features developed, and no pathogenic variants were detected by genetic testing. Initial cardiac catheterization demonstrated preserved pulmonary vasoreactivity and hemodynamics compatible with surgical intervention. Consequently, the patient underwent pulmonary artery banding to reduce pulmonary overcirculation. However, despite surgical intervention and subsequent medical therapy, pulmonary vascular resistance and pulmonary hypertension continued to progress. Lung transplantation was ultimately required. This clinical course demonstrates that PVOD diagnosis is challenging in patients with CHD and that preserved vasoreactivity and apparently operable hemodynamics do not necessarily exclude underlying PVOD. Retrospective review of the initial evaluation did not reveal radiographic or clinical features suggestive of PVOD. Although chest computed tomography showed no clear characteristic findings, assessment of subtle abnormalities was limited because the patient was unable to suspend respiration.

The role of pulmonary vasodilator therapy in PVOD remains controversial. It may increase blood flow in an obstructed pulmonary venous circulation and potentially precipitate pulmonary edema.^6^, ^7^, ^8^ Consequently, pulmonary edema development following pulmonary vasodilator therapy initiation has traditionally been regarded as a clue to PVOD diagnosis. However, selected patients have been recently reported to tolerate pulmonary vasodilators under careful monitoring. Nakamura et al^9^ noted that pulmonary edema developed in only a subset of patients with PVOD receiving pulmonary vasodilator therapy. Similarly, patients with CHD-associated PVOD/PCH have not been universally known to develop pulmonary edema. In the present case, tadalafil, macitentan, and prostacyclin were administered because of maintained pulmonary vasoreactivity; however, pulmonary hypertension continued to progress despite the patient receiving combination therapy. Notably, clinically significant pulmonary edema did not develop. Its absence may have reduced clinical suspicion for PVOD, contributing to the delayed diagnosis.

Histopathologically, the explanted lungs showed diffuse fibrous intimal thickening of the pulmonary veins and venules with prominent hemosiderin deposition, while advanced pulmonary arterial remodeling was limited. Therefore, pulmonary venous pathology was shown to be the predominant mechanism underlying the patient's progressive pulmonary hypertension. According to the 2022 Japanese Clinical Practice Guidelines for PVOD/PCH, subsegmental perfusion defects or normal findings on lung perfusion scintigraphy are the characteristic imaging features of PVOD/PCH.^10^ Consistent with these guideline-described findings, our patient had multiple bilateral subsegmental perfusion defects detected by lung perfusion scintigraphy. Furthermore, the distribution of perfusion abnormalities corresponded to the heterogeneous distribution of pulmonary venous occlusive lesions identified in the explanted lungs. The clinical significance of this observation remains uncertain; however, these findings suggest that lung perfusion scintigraphy may reflect the regional distribution of pulmonary venous disease. Quantitative pathological assessment demonstrated a heterogeneous lobar distribution of pulmonary venous occlusive lesions, with the highest lesion density in the left upper lobe, where the most prominent perfusion abnormality was also observed. Although the clinical significance and generalizability of this observation remain uncertain, the unexplained subsegmental perfusion defects may have provided a clue to the diagnosis of PVOD/PCH in this patient. Although not diagnostic of PVOD/PCH, unexplained subsegmental perfusion defects may serve as a useful clue prompting consideration of PVOD/PCH in patients with unexplained or atypical pulmonary hypertension.

## Conclusions

This case emphasizes the diagnostic challenge of PVOD in a child with a VSD. Although pulmonary hypertension was initially attributed to CHD-associated increased pulmonary blood flow, the subsequent progressive and treatment-resistant clinical course was ultimately explained by underlying PVOD. Therefore, clinicians should consider PVOD in the differential diagnosis of patients with CHD whose pulmonary hypertension persists despite appropriate medical and surgical management. Lung transplantation remains the only definitive treatment for advanced PVOD; thus, early recognition and timely referral to specialized centers are crucial.

## Funding Support and Author Disclosures

The authors have reported that they have no relationships relevant to the contents of this paper to disclose.

**Take-Home Messages**

• PVOD should be considered in children with congenital heart disease who develop progressive pulmonary hypertension despite apparently favorable hemodynamics and preserved vasoreactivity at initial evaluation.
• The absence of pulmonary edema during pulmonary vasodilator therapy does not exclude PVOD and may contribute to delayed diagnosis.
