# Supplementary material for: Pulmonary Veno-Occlusive Disease Presenting as Congenital Heart Disease–Associated Pulmonary Hypertension
Source: JACC Case Rep. 2026 Aug 20;31(40):109910. doi: 10.1016/j.jaccas.2026.109910 (PMC13669881; doi:10.1016/j.jaccas.2026.109910)
Supplement: Supplementary file 1 [file mmc1.docx]

**Supplemental Table 1**. Quantitative assessment of pulmonary venous occlusive lesions

| **Lung lobe** | **Number of lesions** | **Evaluated tissue area, cm²** | **Lesion density, lesions/cm²** |
| --- | --- | --- | --- |
| Right upper lobe | 192 | 15.94 | 12.04 |
| Right lower lobe | 75 | 16.48 | 4.55 |
| Left upper lobe | 357 | 18.09 | 19.73 |
| Left lower lobe | 132 | 15.15 | 8.71 |

Lesion density was calculated as the number of pulmonary venous occlusive lesions divided by the evaluated tissue area.
